# *CHEK2^p.I157T^* Mutation Is Associated with Increased Risk of Adult-Type Ovarian Granulosa Cell Tumors

**DOI:** 10.3390/cancers14051208

**Published:** 2022-02-25

**Authors:** Peter Švajdler, Peter Vasovčák, Marián Švajdler, Monika Šedivcová, Veronika Urbán, Michal Michal, Roman Mezencev

**Affiliations:** 1 Cytopathos s. r. o., 831 03 Bratislava, Slovakia; svajdlerp@cytopathos.sk; 2 Agel Nový Jíčín, a.s., 741 01 Nový Jíčín, Czech Republic; peter.vasovcak@lab.agel.cz; 3 Šikl’s Department of Pathology, Charles University in Prague, Faculty of Medicine and Faculty Hospital in Pilsen, 301 00 Pilsen, Czech Republic; michal@medima.cz; 4 Bioptická Laboratoř s. r. o., 326 00 Pilsen, Czech Republic; sedivcova@biopticka.cz; 5 National Cancer Institute, 833 10 Bratislava, Slovakia; veronika.urban@nou.sk; 6 Georgia Institute of Technology, School of Biological Sciences, Atlanta, GA 30332, USA

**Keywords:** *CHEK2*, *FOXL2*, granulosa cell tumor, adult-type granulosa cell tumor, ovarian cancer, I157T, 1100delC, inhibin, calretinin, SF1

## Abstract

**Simple Summary:**

Granulosa cell tumors of the ovary represent a distinct subset of ovarian cancers typically characterized by hormonal disbalance, slow disease progression, and late recurrence years after surgical removal of the primary tumor. Risk factors associated with development of these rare tumors have not yet been established. In this study, we identified an association between increased risk of developing adult-type granulosa cell tumors (AGCTs) and a specific germline mutation in the *CHEK2* gene. Our findings further support the relevance of this deleterious mutation in the increased risk of various cancer types, and opens a new avenue that can be exploited for future development of CHEK2-targeted preventive and therapeutic interventions directed at AGCTs.

**Abstract:**

Pathogenic germline mutations c.1100delC and p.I157T in the *CHEK2* gene have been associated with increased risk of breast, colon, kidney, prostate, and thyroid cancers; however, no associations have yet been identified between these two most common European founder mutations of the *CHEK2* gene and ovarian cancers of any type. Our review of 78 female heterozygous carriers of these mutations (age > 18 years) found strikingly higher proportion of adult-type granulosa cell tumors of the ovary (AGCTs) among ovarian cancers that developed in these women (~36%) compared to women from the general population (1.3%). Based on this finding, we performed a cross-sectional study that included 93 cases previously diagnosed with granulosa cell tumors, refined and validated their AGCT diagnosis through an IHC study, determined their status for the two *CHEK2* mutations, and compared the prevalence of these mutations in the AGCT cases and reference populations. The prevalence ratios for the p.I157T mutation in the AGCT group relative to the global (PR = 26.52; CI95: 12.55–56.03) and European non-Finnish populations (PR = 24.55; CI95: 11.60–51.97) support an association between the *CHEK2^p.I157T^* mutation and AGCTs. These rare gynecologic tumors have not been previously associated with known risk factors and genetic predispositions. Furthermore, our results support the importance of the determination of the *FOXL2^p.C134W^* somatic mutation for accurate diagnosis of AGCTs and suggest a combination of IHC markers that can serve as a surrogate diagnostic marker to infer the mutational status of this *FOXL2* allele.

## 1. Introduction

Checkpoint kinase 2 is a serine/threonine protein kinase encoded by the *CHEK2* gene involved in cellular responses to genotoxic stress. Depending on the cell context and the DNA damage extent, responses mediated by the CHEK2 kinase can include cell cycle checkpoint activation, DNA repair, DNA damage tolerance, cell senescence, or apoptosis (reviewed in [1]).

Pathogenic germline mutations in the *CHEK2* gene have been consistently associated with mildly to moderately increased risk of developing cancers of the female breast, prostate, and kidney, and some evidence also supports increased risk of developing colorectal cancers, papillary thyroid carcinoma, melanoma, endometrial, testicular, and male breast cancers, as well as some leukemias and lymphomas (reviewed in [2]). Because of numerous recognized *CHEK2* sequence variants with different phenotypic effects, and due to its expression in a variety of tissues, the full scope of cancers associated with inactivating mutations of *CHEK2* has yet to be determined [3].

Truncating c.1100delC (p.T367fs*15) and missense p.I157T (c.470T>C) are the two most common European founder mutations of *CHEK2*. Depending on the populations studied, they have been shown to increase risk for breast and colorectal cancers twofold (c.1100delC) and the risk of breast, colon, kidney, prostate, and thyroid cancers 1.5–4.5-fold (p.I157T) [4]. In addition, c.100delC heterozygosity has been associated with even higher risk of breast cancer in women with a family history of breast cancer [5], but also with a younger age of breast cancer onset [5], increased risk of bilateral breast cancers [6,7], and worse prognosis among women with estrogen receptor (ER)-positive cases [6]. In contrast, however, the missense p.I157T mutation has not been associated with significantly lower age at onset or worse prognosis of breast cancer patients, which implies a possible difference between these two mutations in their functional consequences [8].

Germline testing for selected *CHEK2* mutations has been included in most breast cancer, colorectal cancer, and pan-cancer panels from major commercial genetic testing laboratories [9].

We have previously reviewed list of 78 female patients (age ≥ 18 years) who received genetic counseling at the Department of Medical Genetics, National Cancer Institute, Bratislava, Slovakia, during the period of 2009–2019 due to personal or family cancer history, who were found to be heterozygous carriers of either the p.I157T (N = 71) or the c.1100delC (N = 7) germline *CHEK2* mutation. Twelve of these patients were diagnosed with ovarian cancer: seven patients had epithelial ovarian cancer (age at diagnosis: 50.5 years), four patients had adult granulosa cell tumor (AGCT) (median age at diagnosis: 47.5 years), and in one case the histological type was unknown (age at diagnosis: 23 years). Since AGCT reportedly represents only 3–5% of all ovarian cancers in the general population [10], the high proportion (36.4%) of this malignancy in this patient cohort is striking. Based on these observations, we hypothesized a positive association between the two most common European founder mutations of *CHEK2* and the risk of developing AGCT.

In this report we present the results of our study that further support the implied association between the *CHEK2* germline mutation p.I157T and the risk of developing AGCTs. In addition, we present the results of our evaluation of four immunohistochemical markers used for the diagnosis of GCTs. We report that FOXL2 is the most sensitive single IHC marker for the diagnosis of AGCTs that also shows a fair concordance with the *FOXL2^p.C134W^* somatic mutation, which is pathognomonic for AGCTs. We suggest a combination of three IHC markers as a surrogate marker for this mutation.

## 2. Materials and Methods

Cases diagnosed with ovarian granulosa cell tumors (GCTs) between 1994 and 2018 in women aged 18 or more years at diagnosis were retrieved from the Tumor Registry at Bioptical Laboratory Pilsen, and the registry of the Šikl’s Department of Pathology in Pilsen, Czech Republic. The most representative paraffin blocks representing primary or metastatic lesions were selected; 4 µm sections were produced and stained by hematoxylin–eosin or processed for evaluation of expression of four selected GCT markers by IHC.

**Figure 1:**
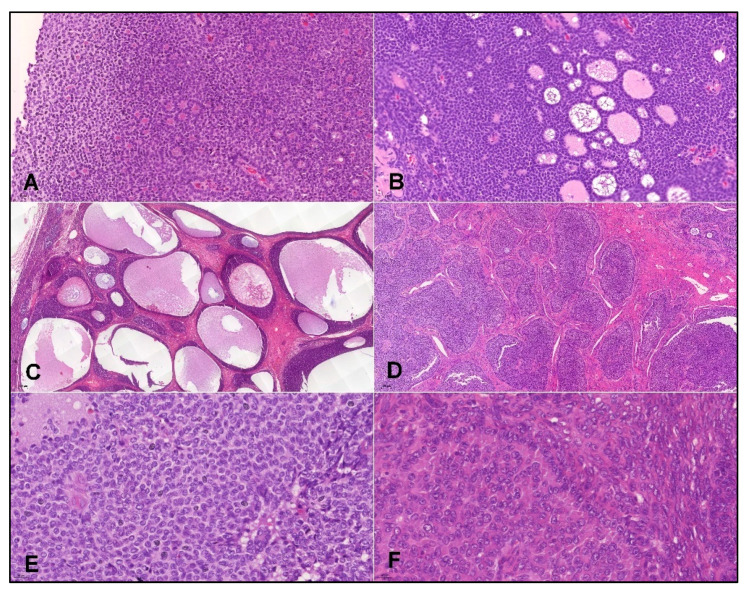
Representative images of HE staining in adult granulosa cell tumors. AGCTs show a variety of histology patterns, including solid, with the presence of: Call–Exner bodies (**A**), original magnification ×200; Microfollicular (**B**), original magnification ×200; Macrofollicular (**C**), original magnification ×50; or insular (**D**), original magnification ×50. Tumor cells have irregular nuclear membranes, oval nuclei, and nuclear grooves (**E**), original magnification ×400. Luteinized granulosa cell tumor cells have abundant eosinophilic cytoplasm and less conspicuous grooving of the nuclei (**F**), original magnification ×400.

Histopathology and IHC:

Hematoxylin–eosin slides from all cases were reviewed to confirm the diagnosis of AGCT. The tumors showed a variety of histology patterns. Diffuse growth pattern was the most common, but other patterns, including trabecular, insular, microfollicular (with the presence of rare Call–Exner bodies), and macrofollicular patterns were also present among these cases. Tumor cells featured scant cytoplasm and uniform oval nuclei with irregular nuclear membranes and nuclear grooves. Luteinization was present in rare cases (Figure 1). In some cases, AGCTs were distinguished from fibromas/thecomas using reticulin stain.

**Figure 2:**
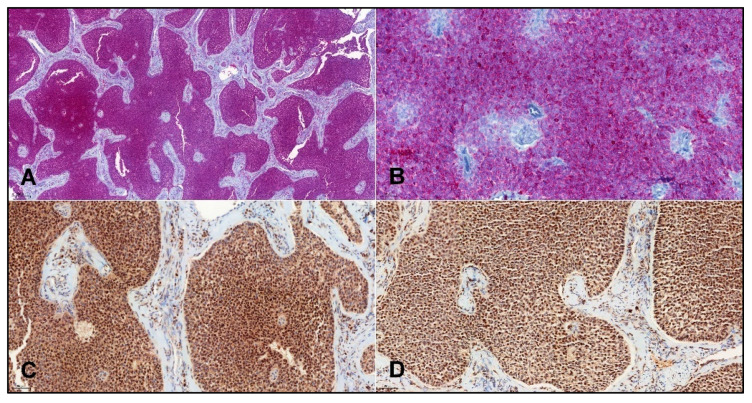
Representative images of IHC staining in adult granulosa cell tumors: Calretinin (**A**), original magnification ×50; inhibin alpha (**B**), original magnification ×200; SF1 (**C**), original magnification ×200; and FOXL-2 (**D**), original magnification ×200.

The immunohistochemical study was performed using the Ventana Benchmark XT automated stainer (Ventana Medical System, Inc., Tucson, AZ, USA). The following primary antibodies were used: Anti-FOXL2 (polyclonal, Invitrogen, Waltham, MA, USA, dilution 1:100), Anti-inhibin alpha (clone R1, CellMarque, Rocklin, CA, USA, ready-to-use), Anti-calretinin (SP65, Ventana, Tucson, AZ, USA, ready-to-use), and anti-SF1 (EPR19744, AbCam, Cambridge, UK, dilution 1:100), using either DAB/HRP (anti-FOXL2, anti-SF1), or Fast Red/ALP (anti-inhibin α, anti-calretinin) as a chromogen/reporter enzyme combination. Appropriate positive and negative controls were used. Immunostaining in >5% of cells was considered positive, and immunostaining in ≤5% of cells was considered negative. Positive results were recorded as focal (staining in >5% and ≤50% of tumor cells) or diffuse (>50%) patterns (Figure 2).

DNA extraction

DNA from formalin-fixed paraffin-embedded (FFPE) tumor tissue was extracted using QIAsymphony DSP DNA Mini Kit (Qiagen, Hilden, Germany) on an automated extraction system (QIAsymphony SP, Qiagen) according to the manufacturer’s supplementary protocol for FFPE samples (Purification of genomic DNA from FFPE tissue using the QIAamp DNA FFPE Tissue Kit and Deparaffinization Solution). Concentration and purity of isolated DNA were determined using NanoDrop ND 1000 (NanoDrop Technologies Inc., Wilmington, DE, USA). DNA integrity was examined by amplification of control genes in a multiplex PCR [11].

Analysis of *FOXL2* and *CHEK2* mutations

The analyses of hot spot mutation c.402C>G, (p.C134W) of the *FOXL2* gene (NCBI RefSeq: NM_023067.4) and two hotspot mutations c.470T>C, (p.I157T) and c.1100delC, (p.T367fs) of the *CHEK2* gene (NCBI RefSeq: NM_007194.4) were performed using PCR and Sanger sequencing. PCR reactions were used for amplification of relevant regions of CHEK2 (part of exon 4 containing c.470T>C; part of exon 11 with c.1100delC) and FOXL2 (part of exon 1 containing c.402C>G). Briefly, 100 ng DNA was added to a reaction mixture consisting of 12.5 µL of FastStart PCR Master (Roche Diagnostic, Mannheim, Germany), 10 pmol of forward and reverse primers (Appendix A, Table A1) and distilled water up to 25 µL. The amplification program: initial denaturation (95 °C for 9 min), followed by 40 cycles of denaturation (95 °C for 1 min), annealing (56 °C (*CHEK2* c.470) or 60 °C (*FOXL2* and *CHEK2* c.1100) for 1 min) and extension (72 °C for 1 min). The program was terminated by incubation at 72 °C for 7 min. The PCR products were separated by electrophoresis on a 2% agarose gel. Successfully amplified PCR products selected for sequencing analysis were purified with magnetic particles Agencourt^®^ AMPure^®^ (Agencourt Bioscience Corporation, A Beckman Coulter Company, Beverly, MA, USA), according to the manufacturer’s protocol, and both sides were sequenced using the Big Dye Terminator Sequencing kit (Applied Biosystems, Waltham, MA, USA) on an automated sequencer ABI Prism 3130xl (Applied Biosystems) at a constant voltage of 13.2 kV for 20 min. The results were analyzed using Geneious 6.1.6 analysis software (Geneious) or visually inspected. DNA sequences were compared to the reference sequence by the online program BLAST [12].

Data analysis and statistics

Cases with focal or diffuse expression of FOXL2, inhibin, calretinin, or SF1 were classified as positive for these IHC markers. The Genome Aggregation Database (gnomAD) [13] was used for retrieval of prevalence of the *CHEK2*^p.I157T^ mutation in three different reference populations. This database aggregates and harmonizes exome and genome sequencing data for 141,456 (v2.1) or 76,156 (v3.1) unrelated individuals from various disease-specific and population genetic studies [14]. Confidence intervals of 95% for proportions were determined using Wilson’s procedure with a correction for continuity implemented in VassarStats [15]. Association between status of the two germline *CHEK2* mutations and group membership (AGCT vs. population reference groups) was assessed from prevalence ratios (PRs) determined using the OpenEpi tool version 3.01 updated 4 June 2013 [16,17]. Confidence intervals of 95% for PRs were approximated by a Taylor series, and the significance of difference from PR = 1 was tested using the Mid-p exact method. The Kaplan––Meier method implemented in GraphPad Prism version 8.0.1 for Windows (GraphPad Software, LaJolla, CA, USA) was used to analyze the time to event (age at AGCT diagnosis) data. Difference between groups of *CHEK2* mutation carriers and non-carriers were assessed using the Gehan–Breslow–Wilcoxon test, which does not require a consistent hazard ratio and gives more weight to the diagnosis of AGCT at an earlier age. Performance of IHC markers for diagnosis of *FOXL2*^p.C134W^ AGCTs was characterized by sensitivity, specificity, and Youden’s J index that summarizes the performance of markers giving equal weight to false positive and false negative values. The 95% confidence intervals for sensitivity and specificity were computed by the Wilson–Brown method using GraphPad Prism version 8.0.1. Youden’s J indices, including CI95 intervals, were calculated using the Two-Way Contingency Table Analysis implemented in StatPages [18].

## 3. Results

We identified 93 cases diagnosed with ovarian granulosa cell tumors from primary (N = 74) or metastatic (N = 19) biopsy specimens. Age at diagnosis was 18–83 years (median = 58 years).

The *FOXL2* mutation p.C134W (c.402C>G) was determined in 69 cases and found in 58 cases (57 cases with heterozygous and one with homozygous genotype), which account for 84.1% (CI95: 72.8–91.4%) of cases with determined status of this mutation. A total of 11 cases were negative and the status of the *FOXL2* could not be determined in 24 cases (Table 1).

### 3.1. Prevalence of CHEK2^p.I157T^ Mutation Is Increased among Adult GCT Patients

The *CHEK2* c.1100delC mutation was found in a single case of a primary ovarian tumor with the *FOXL2*^p.C134W^ somatic mutation, morphologically consistent with AGCT and positive by IHC for inhibin, FOXL2, calretinin, and SF1. However, this mutation was not further considered in the context of its association with AGCTs due to an insufficient number of cases in our group.

For the analysis of the association of *CHEK2* founder mutations with GCTs, we only included in the analysis 58 cases with tumors positive for the *FOXL2* mutation p.C134W, which is pathognomonic for adult-type GCTs (Table 1).

The group with the *FOXL2*^p.C134W^ mutation displays higher median age at diagnosis than the group negative for this mutation (*FOXL2* wild-type). In addition, our *FOXL2*^p.C134W^-positive group includes only cases with a minimum age at diagnosis of 28 years, while the *FOXL2* wild-type group includes also women diagnosed at a younger age. As a result, the *FOXL2*^p.C134W^-positive group largely represents cases of bona fide adult-type ovarian granulosa cell tumor (AGCTs).

Among the 46 cases with known status for the *CHEK2^p.I157T^* germline mutation, six patients were found to be carriers of this mutation (Table 1), which indicates the prevalence of 13.0% (CI95: 5.4–27.0%). The Genome Aggregation Databases v.2.1.1 and v.3.1.1 indicate global prevalence of the *CHEK2^p.I157T^* mutation as 1391/282,816 (0.49%; CI95: 0.46–0.52%) and 615/152,156 (0.40%; CI95: 0.37–0.43%), respectively. Prevalence for the European non-Finnish populations was found to be 686/129140 (0.53%; CI95: 0.49–0.57%) and 362/68,034 (0.53%; CI95: 0.48–0.59), respectively. The highest prevalence of the *CHEK2^p.I157T^* mutation was found for the Finnish population 627/25,118 (2.50%; CI95: 2.31–2.70%) and 245/10,606 (2.31%; CI95: 2.04–2.62%). Our results indicate significantly higher prevalence of the *CHEK2^p.I157T^* mutation among patients with AGCTs than in the global population (*p* = 1.2 × 10^−7^), the European non-Finnish population (*p* = 1.9 × 10^−7^), or the European Finnish population (*p* = 0.0011). Prevalence ratios for the p.I157T mutation in the AGCT group were PR = 26.52 (CI95:12.55–56.03), PR = 24.55 (CI95: 11.60–51.97), and PR = 5.23 (CI95: 2.47–11.06) relative to the global, European non-Finnish, and European Finnish populations, respectively. Of note, this analysis compared the p.I157 mutation prevalence in our GCT group with different reference populations from the gnomAD database v2.1.

**Figure 3:**
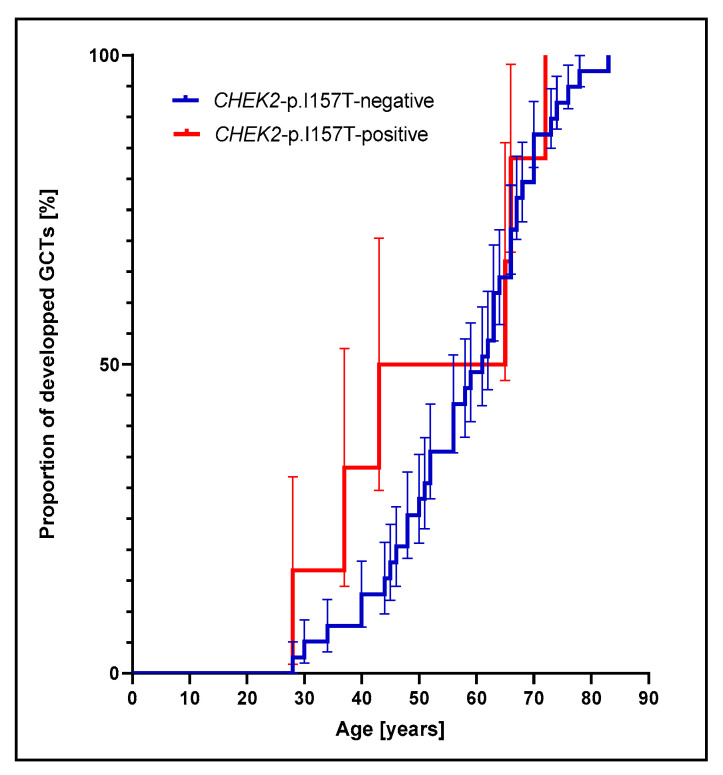
Kaplan–Meier estimate for age at diagnosis of GCT for *CHEK2^p.I157T^*-positive and -negative groups. Percent ovarian GCT cases developed by specific age. Error bars: standard errors (asymmetrical).

Carriers of the *CHEK2^p.I157T^* mutation also displayed a lower median age at diagnosis of the AGCT compared to non-carriers (54 years vs. 60 years). Nevertheless, the difference between the groups was not significant (Gehan–Breslow–Wilcoxon test *p* = 0.4288). However, the log-rank hazard ratio HR = 1.40 (CI95: 0.52–3.78) between the groups with and without the *CHEK2^p.I157T^* mutation is inconclusive, and the Kaplan–Meier analysis does not support the proportional hazards model (Figure 3). When both CHEK2 founder mutations are considered, mutations carriers’ median age at diagnosis is 43 years and thus lower than the median age at diagnosis of 58.5 years in patients with confirmed absence of both founder mutations; however, the difference did not reach statistical significance (Gehan–Breslow–Wilcoxon test *p* = 0.2067).

### 3.2. Performance of IHC Markers for Detection of Adult GCTs

The four selected IHC markers displayed positive or focally positive staining in the following proportions of biopsy specimens: FOXL2: 83/93 cases (89.3%; CI95: 80.7–94.4%); SF1: 77/93 cases (82.8%; 73.3–89.6%); inhibin: 76/93 cases (81.7%; CI95: 72.1–88.7%), and calretinin: 76/93 cases (81.7%; CI95: 72.1–88.7%) (Table 1).

Since the *FOXL2^p.C134W^* (c.402C>G) somatic mutation is pathognomonic for adult-type ovarian granulosa cell tumors (AGCTs), among which it reportedly displays a prevalence of 97% [19], we were interested in exploring the potential of the four typically used IHC markers for GCTs to serve as surrogate markers predicting the presence of this mutation. Positivity for all the four IHC markers was found in 37/58 (63.8%, CI95: 50.1–75.7%) cases with the *FOXL2^p.C134W^* mutation, but only in 3/11 (27.3%, CI95: 7.3–60.7%) cases without this mutation (Fisher’s exact test *p* = 0.0427). In contrast, negative IHC for two or more markers was found in 5/11 (45.5%, CI95: 18.1–75.4%) tumors without the *FOXL2* mutation, but only in 5/58 (8.6%, CI95: 3.2–19.7%) tumors with the *FOXL2* mutation (Fisher’s exact *p* = 0.0068).

The potential of the four IHC markers to serve as surrogate markers for the *FOXL2^p.C134W^* mutation was assessed individually across 69 specimens with a known mutation status of this *FOXL2* allele. The highest sensitivity was found for FoxL2 immunoexpression (~95%) and the highest specificity for inhibin and calretinin (~46%) (Table 2). FOXL2 immunoexpression was found to be in fair agreement with *FOXL2^p.C134W^* mutation status (~84% agreement; Cohen’s kappa = 0.271; CI95: −0.037–0.579).

Association between the expression of all the four IHC markers and *FOXL2^p.C134W^* mutational status was also examined by binary logistic regression. The strongest predictor of *FOXL2* mutation status was FOXL2 immunoexpression, recording an odds ratio of 12.44, controlling for immunoexpression of inhibin, calretinin, and SF1 (Table 3). The logistic model with all four markers (Model 1) displays better data fit than an alternative model with no IHC markers (omnibus test of model coefficients *p* = 0.009); however, its estimated sensitivity 96.6% (CI95: 88.3–99.4%), specificity 27.3% (CI95: 9.7–56.6%) and Youden’s J index (0.238; CI95: 0.010–0.414) are not significantly better than the sensitivity, specificity and Youden’s J index of a single FOXL2 IHC marker (Table 2).

A logistic regression model was also built with three IHC markers FOXL2, inhibin, and calretinin (Model 2, Table 3). These IHC markers were included in Model 2, because they were found to be the most sensitive or most specific predictors of *CHEK2* mutation status as single IHC markers, and their Wald *p*-values in Model 1 were *p* < 0.1. In Model 2, FOXL2 expression remained the strongest predictor of *FOXL2^p.C134W^* mutation with an odds ratio of 10.61, controlling for inhibin and calretinin. This model with three predictors displayed sensitivity 94.8% (CI95: 90.1–98.4%), specificity 45.5% (CI95: 20.4–64.5%), and Youden’s J index 0.403 (0.104–0.629) (Table 3), and it performed better than the logistic Model 1 that included all four IHC markers, although the difference in classification was not statistically significant for our dataset (McNemar’s test *p* = 0.25). The model with three predictors performed better than any single IHC marker (Table 2), but the differences were also not statistically significant (McNemar’s test *p* > 0.05).

When the analysis was limited to 58 cases positive for *FOXL2^p.C134W^* mutation, FOXL2 expression was positive in 94.8% cases (CI95: 85.9–98.6%), inhibin in 86.2% (CI95: 75.1–92.8%), calretinin in 86.2% (CI95: 75.1–92.8%), and SF1 in 84.5% (CI95: 73.1–91.6%) (Table 2). No agreement above chance was found between immunostaining by FOXL2 and calretinin (Cohen’s kappa = −0.068; CI95: −0.126–(−0.009)).

The highest agreement was found between immunostaining by inhibin and SF1 (Cohen’s kappa = 0.262; CI95: −0.055–0.580), even though the degree of agreement was inconclusive due to the small sample size and a wide 95% confidence interval. Taken together, SF1 appears to be a redundant IHC marker providing lower performance as well as limited additional information compared to inhibin for the prediction of *FOXL2* mutation status.

For the remaining pairs of IHC markers, low values of estimated Cohen’s kappa imply only slight agreement between these markers; however, wide confidence intervals do not allow for drawing confident conclusions: calretinin and inhibin (Cohen’s kappa = 0.130; CI95: −0.180–0.440); FOXL2 and inhibin (Cohen’s kappa = 0.115; CI95: −0.196–0.426); calretinin and SF1 (Cohen’s kappa: 0.105; CI95: −0.193–0.402); and FOXL2 and SF1 (Cohen’s kappa = 0.0966; CI95: −0.190–0.329).

## 4. Discussion

Ovarian granulosa cell tumors (GCTs) are rare gynecologic tumors that represent less than 5% of all ovarian tumors [20]. They account for about 70% of all sex cord-stromal tumors (SCST) that arise from the gonadal primitive sex cords or stromal cells.

Adult-type ovarian granulosa cell tumors (AGCTs) and juvenile-type ovarian granulosa cell tumors (JGCTs) are epidemiologically, clinically, and histopathologically distinct entities that account for 95% and 5% of GCTs, respectively [21]. AGCTs are typically diagnosed in perimenopausal or early postmenopausal women with a median age at diagnosis of 50–54 years, although they can occasionally also be diagnosed in children [22]. In contrast, the average age at diagnosis of JGCT is reportedly 13 years, although 21% of cases were still diagnosed in women over 21 years of age [23]. Of note, these cases were typically classified as AGCTs or JGCTs based on morphological criteria.

Consistent with previous reports [22], we found the median age at diagnosis of ovarian GCTs to be 58 years among all patients and 59 years in a subgroup with the *FOXL2^p.C134W^* mutation. On the other hand, a Korean case-series study of 91 patients with adult-type ovarian GCT reported the median age at diagnosis to be 42 years (range 7–85 years) [24], which is considerably lower than was found in our study or reported by other investigators. This disagreement may be caused by different definitions of AGCT, which is molecular in our study and morphological/clinical in the Korean study. Relatively non-specific histopathologic features of AGCTs have been recognized as a source of misdiagnosis of other cancers as AGCTs and imply the importance of molecularly defined diagnosis of these tumors [25].

The presence of the somatic mutation *FOXL2^p.C134W^* and the expression of FOXL2 protein are characteristic of the adult-type GCT. In contrast, juvenile-type GCTs virtually never display this mutation, and the expression of FOXL2 may be variable, and even reduced in aggressive phenotypes and advanced stages of JGCTs (reviewed in [26]). Ovarian granulosa tumors with the *FOXL2^p.C134W^* mutation reportedly displayed higher expression of FOXL2 on mRNA level than those with wild-type *FOXL2*, which in turn correlated with the intensity of FOXL2 IHC staining among AGCTs and JGCTs [21]. Consistent with these findings, our results show more prevalent positivity of FOXL2 IHC among cases with the *FOXL2^p.C134W^* mutation than in cases without this mutation (PR = 1.3; CI95:0.90–1.88). We found this association between the *FOXL2^p.C134W^* mutation status and FOXL2 positivity by IHC to be marginally statistically significant (mid-*p*-value = 0.052).

Consequently, the presence of somatic mutation *FOXL2^p.C134W^* can serve as a defining feature of the AGCTs; therefore, in this study we delineated the AGCTs as cases previously diagnosed with ovarian granulosa cell tumors or granulosa cell-like tumors, in which we additionally found the somatic mutation *FOXL2^p.C134W^*. This approach is in line with that of other investigators, who reappraised *FOXL2* wild-type AGCT cases, which were previously diagnosed entirely by morphology, as most likely representing thecomas or fibromas [19,26,27]. It should be noted, however, that *FOXL2^p.C134W^* was also detected in a subset of thecomas [19].

To streamline the diagnosis of AGCTs, we evaluated the performance of selected IHC markers to distinguish ovarian granulosa cell tumors with the *FOXL2^p.C134W^* mutation from granulosa cell tumors with wild-type *FOXL2*, as the latter cases presumably represent JGCTs and possibly some misdiagnosed SCSTs of other types. Our results show a generally appreciable sensitivity but relatively low specificity of individual IHC markers.

The best diagnostic performance was achieved using a logistic regression model that integrated IHC expression of FOXL2, inhibin, and calretinin. However, due to a relatively small sample size, diagnostic performances were not found to be significantly different across several classifiers discussed in this study. The problem of small sample size affects most studies reporting research on this rare type of ovarian cancer. Nevertheless, research on rare cancers, such as gastrointestinal stromal tumors, acute myeloid leukemia, seminomas, and others, produced fundamental insights translatable into innovative therapeutic strategies for these rare malignancies, as well as in better a molecular understanding of more common types of cancers [28]. This is because rare cancers are usually homogeneous entities that (i) tend to result from a single identifiable genetic cause or exposure to a single identifiable environmental carcinogen, (ii) can be characterized by a small number of mutations, and (iii) typically deviate from normal cells only in a small number of pathways that are amenable to therapeutic targeting.

Thus far, no reproductive, occupational, environmental, or general lifestyle risk factors have been consistently associated with the risk of developing AGCTs [29], and no inherited predispositions have been identified for the development of these tumors. For these reasons, AGCTs have been considered sporadic and unrelated to exposure.

The study presented in this report was motivated by our finding of an unexpectedly high prevalence of AGCTs among female carriers of the *CHEK2^p.I157T^* mutations diagnosed with ovarian cancer. AGCT reportedly represents only 3–5% of all ovarian cancers in the general population [10]. Among the 75,024 ovarian cancers (ICD-O-3 site code C56.9) registered in the US SEER 13 cancer registries over 1992–2018, only 974 cases represented GCTs (ICD-O-3 code 8620/3), which accounted for 1.3% of all ovarian cancers [30]. By contrast, however, GCTs represented 4 of 11 (~36.4%) histologically characterized ovarian cancers in a group of the *CHEK2^p.I157T^* mutation carriers.

Our results further demonstrated a higher prevalence of this germline mutation among AGCT patients relative to three reference populations, which provided support to our hypothesis of the positive association between the *CHEK2^p.I157T^* mutation and adult-type ovarian granulosa cell tumors. Since GCTs are rare tumors that represent no more than 5% of all ovarian malignancies and display a similar occurrence across various populations [10], the risk of bias potentially generated by the selection of reference populations is low. Notably, we also found a higher prevalence of the *CHEK2^p.I157T^* mutation in the group of AGCT patients than in the European Finnish population that displays the highest prevalence of this mutation (Ensembl, release 105 [31]; rs17879961), which further supports our conclusion.

Our study implicated for the first time the *CHEK2^p.I157T^* mutation in granulosa cell carcinogenesis and suggested a specific genetic predisposition in adult-type ovarian granulosa cell tumors. The *CHEK2^p.I157T^* mutation has been previously shown to impede Chek2 protein homodimerization, which is required for its activation, and to interfere with wild-type Chek2 protein in heterozygous cells in a dominant-negative manner [8]. This mutation is considered to confer multi-organ tumor susceptibility through its probable synergism with other genetic factors or environmental exposures [3]; however, no conclusive association has been reported between this mutation and any type of ovarian cancer thus far. A small case-control study of patients with GCT suggested an association with family history of breast (OR = 2.13; 1.19–3.80) or ovarian cancer (OR = 2.89; 1.08–7.72), which implies a possible existence of shared genetic predispositions between these cancers [32].

An underlying mechanism behind the role of the *CHEK2^p.I157T^* germline mutation in the potentially increased risk of AGCTs has yet to be determined. One possible explanation can be that this germline *CHEK2* mutation, which affects cell cycle regulation and DNA damage response pathways, can predispose to unique patterns of subsequent somatic mutations, including *FoxL2^p.C134W^*, which is pathognomonic for AGCTs. A similar mechanism has been previously suggested for prostate cancer, since patients with pathogenic germline *CHEK2* mutations displayed a significantly higher prevalence of the somatic *CDK12* mutation than unselected prostate cancer patients from the TCGA cohort. Consequently, *CHEK2* germline mutations have already been associated with increased occurrence of a specific somatic mutation in a cancer-relevant gene [33].

Besides this suggested role of the germline *CHEK2^p.I157T^* mutation in the risk of developing a subsequent *FOXL2^p.C134W^* mutation in granulosa cells, other mechanisms can also be operative in the AGCT-*CHEK2^p.I157T^* association identified in our study. For instance, a wild-type *FOXL2* was shown to modulate cell cycle regulators and promote cell cycle arrest in the G1 phase and in the repair of oxidatively damaged DNA [34]. This implies that the presence of the germline *CHEK2* mutation impairing the cell cycle and DNA damage response may synergize with the *FOXL2^p.C134W^* mutation that may develop in granulosa cells as a “second hit” and further aggravate the impairment of the cell cycle and DNA damage repair processes, paving the way for ovarian granulosa carcinogenesis. Nevertheless, these suggested mechanisms are not mutually exclusive and can both contribute to the development of granulosa cell tumors.

## 5. Conclusions

The *CHEK2* missense p.I157T (c.470T>C) germline mutation, which was previously reported to increase the risk of breast, colon, kidney, prostate, and thyroid cancers [4], is also associated with adult granulosa cell tumors (AGCTs). This association suggests that *CHEK2^p.I157T^* can be a predisposing genetic factor for AGCTs.

The *FOXL2^p.C134W^* mutation, which is a pathognomonic defining feature of AGCTs, was detected among ~84% of cases previously diagnosed as AGCTs based on clinical and histopathological findings. This finding supports the necessity of including detection of the *FOXL2^p.C134W^* mutation in the diagnosis of AGCT. The presence of this mutation is in fair agreement with IHC positivity of the tumor cells for FOXL2 expression, which allows for application of the FOXL2 expression as a surrogate IHC marker for the *FOXL2^p.C134W^* mutation.

Combination of IHC markers FOXL2, inhibin, and calretinin displayed the best performance for the prediction of *FOXL2^p.C134W^* mutational status among the AGCT cases diagnosed by clinical and histopathological findings.

## Figures and Tables

**Table 1:** Characteristics of cases diagnosed with ovarian granulosa cell tumors classified per status of the FOXL2^p.C134W^ mutation.

	*FOXL2* Status			
	*FOXL2^p.C134W^*	*FOXL2* Wild-Type	Unknown	ALL
**Number of cases**	58	11	24	93
**Age at diagnosis (years)**				
Median (range)	59 (28–83)	52 (18–81)	59.5 (32–75)	-
***CHEK2* mutation p.I157T**				
Positive	6	1	0	7
Negative	40	6	0	46
Unknown	12	4	24	40
***CHEK2* mutation c.1100delC**				
Positive	1	0	0	1
Negative	39	8	8	55
Unknown	18	3	16	37
**FOXL2 by IHC**				
Positive	55	8	20	83
Negative	3	3	4	10
**Inhibin by IHC**				
Positive	50	6	20	76
Negative	8	5	4	17
**Calretinin by IHC**				
Positive	50	6	20	76
Negative	8	5	4	17
**SF1 by IHC**				
Positive	49	8	20	77
Negative	9	3	4	16

**Table 2:** Performance of IHC markers to predict *FOXL2^p.C134W^* or *FOXL2* wild-type status in tumors diagnosed as GCTs/likely GCTs.

	FOXL2	Inhibin	Calretinin	SF1
**Sensitivity** **(CI95)**	94.8%	86.2%	86.2%	84.5%
	(85.9–98.6%)	(75.1–92.8%)	(75.1–92.8%)	(73.1–91.6%)
**Specificity** **(CI95)**	27.3%	45.5%	45.5%	27.3%
	(9.7–56.6%)	(21.3–72.0%)	(21.3–72.0%)	(9.7–56.6%)
**Youden’s J** **(CI95)**	0.221	0.317	0.317	0.118
	(−0.009–0.448)	(0.008–0.631)	(0.008–0.631)	(−0.116–0.455)

**Table 3:** Logistic regression analysis of association between *FOXL2* mutational status and four IHC markers.

Model Variables (Model 1 and Model 2)								
	B *	SE	Wald	Df	*p*-Value	OR	95% CI for OR	
							Lower	Upper
**Constant**								
Model 1	−2.24	1.22	3.38	1	0.066	-	-	-
Model 2	−2.41	1.21	3.97	1	0.046	-	-	-
**Inhibin**								
Model 1	1.61	0.86	3.47	1	0.062	5.00	0.92	27.13
Model 2	1.44	0.82	3.03	1	0.082	4.20	0.84	21.15
**FOXL2**								
Model 1	2.52	1.02	6.17	1	0.013	12.44	1.70	90.94
Model 2	2.36	1.00	5.63	1	0.018	10.61	1.51	74.63
**SF1**								
Model 1	−0.67	1.05	0.42	1	0.519	0.510	0.066	3.956
Model 2	NA	NA	NA	NA	NA	NA	NA	NA
**Calretinin**								
Model 1	1.61	0.86	3.47	1	0.062	5.00	0.920	27.131
Model 2	1.44	0.82	3.03	1	0.082	4.20	0.835	21.149
**Model fit and classification performance**								
**Omnibus test of model coefficients**		**Chi-squared**			**Df**	***p*-value**		
Model 1		13.51			4	0.009		
Model 2		13.07			3	0.004		
**HL test**		**Chi-squared**			**Df**	***p*-value**		
Model 1		1.13			3	0.771		
Model 2		1.74			2	0.419		
**-2 Log likelihood**		**Chi-squared**			**Df**	***p*-value**		
Model 1		47.03			NA	NA		
Model 2		47.47			NA	NA		
**Classification results**		***FOXL2^p.C134W^***			***FOXL2wt***	***FOXL2^p.C134W^/FOXL2wt***		
Model 1	Correct	56 (96.6%)			3 (27.3%)	59 (85.5%)		
	Incorrect	2			8	10		
	Youden’s J	0.238 (CI95: 0.010–0.414)						
Model 2	Correct	55 (94.8%)			5 (45.5%)	60 (87.0%)		
	Incorrect	3			6	9		
	Youden’s J	0.403 (CI95: 0.104–0.629)						

* Regression coefficient.

## Data Availability

Publicly available datasets analyzed in this study can be found here: gnomAD: [https://gnomad.broadinstitute.org, accessed on 22 October 2021]; SEER*Stat: [https://seer.cancer.gov/data-software/documentation/seerstat/nov2020/, accessed on 22 October 2021]. Other data available on request from the corresponding authors.

## Appendix A

**Table A1:** Primers used for PCR amplification and sequencing of the *FOXL2* and *CHEK2* genes.

Primer Name	Sequence 5′-3′
FOXL2-402-F1	CCTCAACGAGTGCTTCATCA
FOXL2-402-R2	GCCGGTAGTTGCCCTTCT
CHEK2 470 F2	CTCTATTTTAGGAAGTGGGTCC
CHEK2 470 R2	TAGTGACAGTGCAATTTCAGAA
CHEK2 1100F	TGTCTTCTTGGACTGGCAGA
CHEK2 1100R	GGGGTTCCACATAAGGTTCTC
